# Relationship of spindle assembly checkpoint fidelity to species body mass, lifespan, and developmental rate

**DOI:** 10.18632/aging.100416

**Published:** 2011-12-26

**Authors:** Antonello Lorenzini, Lauren S. Fink, Thomas Stamato, Claudio Torres, Christian Sell

**Affiliations:** ^1^ University of Bologna; Department of Biochemistry “G. Moruzzi”; Bologna, Italy; ^2^ Drexel University College of Medicine; Department of Pathology and Laboratory Medicine; Philadelphia, PA 19129, USA; ^3^ Current address; Fox Chase Cancer Center; Department of Cancer Biology; Philadelphia, PA 19046, USA; ^4^ Lankenau Institute for Medical Research; Wynnewood, PA 19096, USA

**Keywords:** tetraploid, mitosis, fibroblasts, mouse, human, stability, genome, lifespan, aging

## Abstract

We have examined the tolerance of the spindle assembly checkpoint (SAC), as measured by the appearance of tetraploid cells in the presence of a microtubule inhibitor, in a series of primary cell strains derived from species with diverse lifespan and body size. We find that the integrity of the SAC varies among these species. There is a robust correlation between the integrity of the SAC and body size, but poor correlation with longevity and parameters of species development (i.e., time of female fertility, gestation length, and postnatal growth rate). The results suggest that fidelity of the SAC co-evolved more closely with the number of mitoses needed to reach adulthood than with species lifespan.

## INTRODUCTION

Rodent and human tissues are histologically indistinguishable, yet the rate of tumor formation differs significantly between the species. A higher rate of transformation in vitro (10^−5^ to 10^−6^ in rodents compared to 10^−9^ to 10^−10^ in humans) and a higher rate of tumor formation in vivo suggest that the rodent genome is inherently less stable [1, 2].

Differences between rodent and human cells have been identified that may underlie differences in genomic stability. For example, the complement of oncogenic changes required for immortalization is greater in human cells than in rodent cells [3], which is potentially linked to differential activation of cell cycle checkpoint proteins such as the Cdk inhibitor p16Ink4a/Arf [4]. DNA repair is essential to genome stability and although DNA repair rates appear similar in rodents and humans [5], variability exists in the levels and activities of essential DNA repair proteins such as the DNA end binding Ku70/80 heterodimer and the DNA activated protein kinase (DNAPK) [5, 6]. We have postulated that the differential expression of these DNA repair proteins provide for a higher fidelity of repair based upon a more stable cell cycle arrest following DNA damage [7, 8].

The spindle assembly checkpoint (SAC) is a critical element in regulating chromosome segregation [9]. This checkpoint is revealed in the presence of microtubule poisons [10] and serves to regulate the E3 ubiquitin ligase anaphase-promoting complex/cyclosome (APC/C) that targets cyclin B to the 26 S proteasome [11]. Twenty years ago, Schimke and co-workers identified a substantial difference in the SAC between rodent and human cells, which may relate to the differential genomic stability in these species [12]. Rodent cells were found to undergo successive rounds of DNA replication in the presence of microtubule inhibitors that prevent mitosis, leading to cells with tetraploid DNA content. More recent work has shown that the SAC can be stabilized in rodent cells through introduction of the human MAD1 protein [13]. These results suggest that enhanced fidelity of the SAC is a feature of longer-lived species contributing to improved genomic integrity.

**Figure 1:**
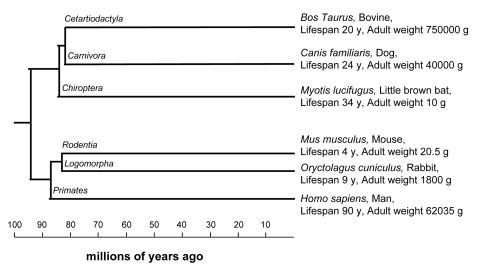
Phylogenetic tree showing relationship among species included in this study Species were chosen from different orders (i.e. having a relatively high evolutionary distance) and so that maximum longevity and adult weight cover a wide range of values without bias towards a single branch. Order names are indicated on branches, species name are shown with relative common name, maximum longevity (expressed in years) and adult weight (expressed in grams), data are from the work of De Magalhaes and co-workers [22]. Phylogenetic relations were from the work of Springer and co-workers [S2].

We have tested this prediction using a series of mammalian cell strains that have been maintained under standardized culture conditions [14]. We chose species belonging to different orders (ie, having a relatively large evolutionary distance) so that maximum longevity and adult weight covered a wide range of values and were not skewed toward a single branch, (see Figure 1). Using fibroblast cells from these species we have examined the relationship between SAC fidelity and maximum longevity, adult weight, time of female fertility, gestation length and postnatal growth rate. It appears that SAC stability correlates better with body size than lifespan, timing of female fecundity, gestation length, or postnatal growth rate. Although the data do not rule out a role for the SAC in maintaining genomic stability required for extended lifespan, our results suggest that SAC has a more prominent role in body size determination.

## RESULTS

**Figure 2:**
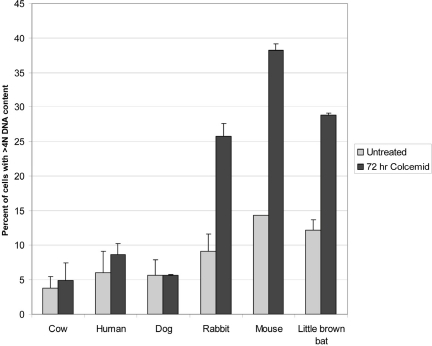
SAC tolerance in the 6 mammalian species analyzed The tolerance (defined as a lack of stringency) of the SAC is graphed as percent of cells with >4N DNA content for the cell strains used in the analysis. Species are ordered by decreasing adult weight from left to right. DNA content was measured as described in materials and methods. Untreated cultures were cells maintained in serum supplemented growth medium as previously described for replicative lifespan studies [14]. Cultures treated with colcemid were maintained for 72 hours in the presence of the inhibitor and DNA content measures at that time. Error bars are standard deviations from one representative experiment of 3 independent replications. Results at 48 hours following colcemid addition fell midway between the values for untreated and 72 hours colcemid treatment while relative differences between species were consistent with the 72 hour results.

In order to examine the relative stability of the SAC in species with differing lifespans, we first confirmed the original observations that rodent cells were unable to maintain a G2 arrest in the presence of a microtubule inhibitor. Because the original observations were made using immortalized cell lines, it is important to verify that primary strains display the same difference in SAC stability. We examined primary fibroblast strains from mouse, dog, rabbit, cow, little brown bat, and human for their ability to maintain the SAC in the presence of the microtubule inhibitor colcemid. Cells were treated with colcemid while in log phase growth and analyzed for DNA content at 48 and 72 hours post-treatment. Flow cytometry analysis indicated, consistent with previous reports [12, 13], that mouse cells were unable to maintain a stable G2 arrest in the presence of colcemid. In addition, we found that the stability of the SAC varied widely across species (Table 1 and Figure 2). Following mitotic block, cells from some species, including mouse, rabbit, and little brown bat, displayed an increased percentage of cells containing greater than 4N DNA content compared with human cells. The increased polyploidy in these cell types in the presence of colcemid suggests that these cells continue to cycle, while human cells remain arrested in mitosis. In contrast, when DNA content was measured in dog and cow fibroblasts following colcemid treatment, there was no significant increase in polyploid DNA content, suggesting that fibroblasts from these species did not progress through the cell cycle.

**Table 1:** Parameters of species analyzed in this study relative to their Spindle assembly checkpoint (SAC) tolerance.*

Species name	Common name	AnAge database reference number *	SAC tolerance in control cells **	SAC tolerance in 72h Colcemid treated cells **	Adult weight	Maximum longevity	Female sexual maturity	Gestation	Postnatal growth rate
(HAGRID)	(>4N DNA % Cells)	(>4N DNA % Cells)	(g)	(years)	(days)	(days)	(days^−1^)
Bos Taurus	Cow	02257	3.8	4.9	750000	20	548	277	0.0031
Canis familiaris	Dog	02422	5.7	5.65	40000	24	510	63	0.0244
Homo sapiens	Human	03116	6	8.67	62035	90***	4745	280	0.0005
Oryctolagus cuniculus	Rabbit	02927	9.1	25.8	1800	9	730	30	0.0228
Myotis lucifugus	Little brown bat	02737	12.2	28.8	10	34	210	55	0.116
Mus musculus	Mouse	03347	14.3	38.2	20.5	4	42	19	0.0298

Notes:

* Adult weight, maximum longevity, female sexual maturity, gestation length and postnatal growth rate are all from the AnAge database [17].

** Species are ordered by increasing SAC tolerance measured as percent of cells with >4N DNA content.

*** Human longevity is here given a value of 90 to account for the fact that human are the only species with a very large available data set and 90 years, more accurately reflects a value for a random relatively small sample of humans.

**Figure 3:**
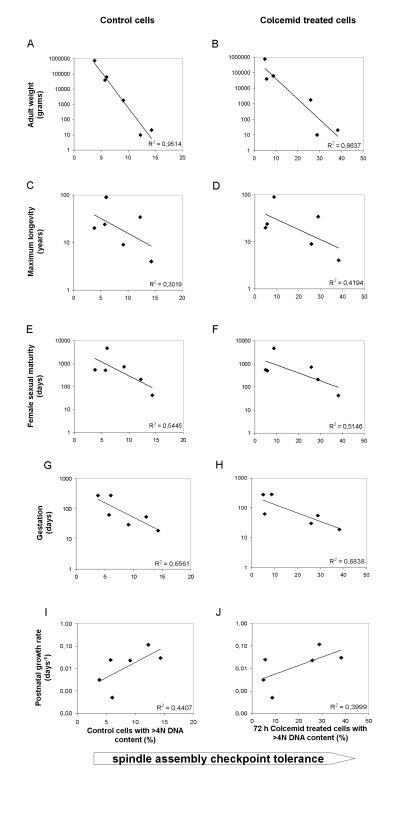
Correlation among the spindle assembly checkpoint (SAC) tolerance with several species parameters The tolerance (defined as a lack of stringency) of the SAC is graphed as percent of cells with >4N DNA content in control cells (panels A, C, E, G, I) or cells treated for 72 h with Colcemid. (panels, B, D, F, H, J). Adult weight, maximum longevity, female sexual maturity, gestation length and postnatal growth rate were all from the AnAge database [22], with the exception of human longevity that was given a value of 90 to account for the fact that human are the only species with a very large available data set and 90 years, which more accurately reflects a value for a random relatively small sample of humans. Measure units are indicated. Postnatal growth rates are expressed in days^−1^ and, as stated in the AnAge database, they were calculated by fitting empirical data taken from published growth curves to Gompertz function. See legend of Figure 2 and methods section for information on cell treatment.

Surprisingly, the ability of fibroblast cultures to stably arrest in mitosis following colcemid treatment did not appear to be associated with species lifespan as we predicted. In order to confirm this observation, we performed regression analysis to determine the strength of the correlation between SAC tolerance, assessed by percentage of cells with >4N DNA content after colcemid treatment, and maximum species lifespan. The correlation between maximum lifespan and polyploid DNA content was poor, with R2 values of 0.3019 and 0.4194 for untreated and colcemid-treated cultures, respectively (Figure 3C and D). Because we previously determined that telomere length in these same fibroblast cultures correlated with body mass rather than lifespan, we used the same regression analysis to analyze body mass and >4N DNA content following treatment with colcemid. We observed a much stronger correlation between polyploidy and body mass than we did with polyploidy and maximum species lifespan (Figure 3A and B; R2 values of 0.9514 and 0.8637 for untreated and colcemid-treated cultures, respectively). Interestingly, the strongest correlation between polyploidy and body size was found in untreated cells (Figure 3A), where the correlation reached 0.9514. To assess for a potential relationship between SAC fidelity and developmental programs, we performed regression analyses between SAC tolerance and female sexual maturity, gestation length, and postnatal growth rate. Although R2 values were higher for each of these parameters than for maximum longevity, in no case was the relative R2 value higher than when SAC tolerance was analyzed in relationship to adult weight (Figure 3).

## DISCUSSION

Consistent with previous work [12], we find that stability of the SAC varies among species and that human cells have a very stable SAC compared with mouse cells. Interestingly, the percentage of cells with ˃4N DNA content, which presumably reflects the inherent stability of the SAC, varies significantly in untreated cells and correlates strongly with the mass of the organism from which the cell strain was derived. The correlation with species lifespan however, is poor, suggesting that the stability of the SAC is related to body mass more strongly than to species lifespan. From an evolutionary viewpoint, it is tempting to speculate that cellular mechanisms ensuring proper cell division may be more relevant for species that require a greater number of mitoses in a relatively short life span, cow for example, while other maintenance mechanisms may be more relevant when fewer mitoses are required over a longer life span, for example in the little brown bat (Figure 1). The high correlation between SAC stringency and species body size suggests that the SAC is more relevant to organisms that require a greater number of cell divisions, i.e., organisms with large body size.

Both polyploid and aneuploid cells accumulate during aging [15, 16] and the development of a tetraploid phenotype may be one stage in the malignant transformation of normal cells. If the mitotic spindle is compromised, cells may arrest at the metaphase-anaphase transition point. The possible escape of cells from this block without proper segregation of sister chromatids and cytokinesis is termed ‘mitotic slippage’. Proliferation of cells that have become tetraploid through mitotic slippage or another mechanism appears to be limited by LATS2-dependent p53 activation [17], which provides a link between the SAC and G1 tetraploidy checkpoint. The loss of p53 or increased levels of negative regulators of p53 such as the MDM2 protein [18] may allow proliferation to occur in tetraploid cells. Thus, it appears that an increased number of tetraploid cells may not be indicative of a greater propensity for tumor development in the absence of deregulation of cell cycle controls. In fact, polyploid cells may occur as part of normal development, generally as a component of terminal differentiation or in response to metabolic stress [19]. It has been suggested that the appearance of polyploid cells may convey an evolutionary advantage in some settings [20]. However, the inverse correlation between the number of polyploid cells and body size suggests that species with a larger body size have developed more stringent controls on mitotic slippage, the most probable source of polyploidy measured in our studies. This argues against an increased percentage of polyploid cells as a mechanism to increase organ or body size and suggests the converse, that species with a large body require a greater stringency in their SAC to prevent unscheduled production of polyploid cells. Polyploid cells can arise through multiple mechanisms including cell fusion, mitotic slippage, or cytokinesis failure [19]. In our experiments, mitotic slippage is the likely cause of polyploid cells. Thus the polyploidy cells observed may represent a response to spontaneous DNA damage, replication fork collapse due to stalling in repetitive regions of the genome, defects in post-replication events such as decatenation leading to SAC activation and mitotic slippage, or a failure in cytokinesis following normal mitosis. Interestingly, persistent telomere damage caused by decreased levels of POT1, a key mediator of telomere maintenance, produces tetraploid cells [21]. However, since these studies were performed in a p53-null background which causes reduced SAC fidelity, the telomere dependent increase in tetraploid may not occur in a p53 competent setting. The fact that the accumulation of polyploid cells correlates with adult weight in untreated cells suggests that limiting the intrinsic rate of formation of polyploid cells is important for species with large body size. Thus, the presence of polyploidy may be more problematic during the accumulation of cells required for a large body size, whereas other aspects of genomic stability are more critical to lifespan. For example, using the same panel of cell cultures, we have recently reported that the G2 checkpoint is more stringent in longer-lived species [7]. We believe that this reflects an enhanced cell cycle checkpoint stability in longer-lived species that functions to ensure a higher fidelity of DNA repair. We have recently proposed that a prolonged “time for repair” could be a fundamental aspect in the soma maintenance machineries necessary to ensure longevity [8]. However, cell cycle checkpoint stringency may be associated with parameters other than lifespan which are characteristic of a particular species. For this reason, we searched for possible correlations of SAC fidelity with some of the available developmental parameters for the species examined.[22] We analyzed the correlation of SAC fidelity with female sexual maturity, gestation length, and postnatal growth rate, and still, the highest correlation remained with adult body mass, the only species parameter that does not include “time”.

In summary, we find that fidelity of the SAC is variable among species and correlates inversely with body size, as evidenced by a lower percentage of polyploid cells in cell cultures from animals with larger body sizes. Surprisingly, the correlation is strongest in cells not treated with colcemid, which suggests that the intrinsic rate of mitotic slippage underlies the correlation. Our findings suggest that SAC fidelity is more critical in species with a large body mass than in species with a long lifespan.

### MATERIALS AND METHODS

#### Species information

Maximum longevity, with the exception of human longevity, adult weight, female sexual maturity, gestation length, and postnatal growth rate were all from the AnAge database [22] (see Table 1). Human longevity was given a value of 90 to normalize for the smaller data sets available in the majority of species. Postnatal growth rates are expressed in days-1 and were calculated by fitting empirical data taken from published growth curves to Gompertz function (Figure S1).

#### Cell culture

Skin fibroblast cultures were maintained as previously described [14]. Briefly, all cultures were maintained using Minimum Essential Medium (MEM) with Earle's salts and L-glutamine (Mediatech; Manassas, VA) containing 10% fetal bovine serum, MEM vitamins, MEM amino acids, and penicillin-streptomycin (Mediatech). Cells were cultivated according to standard procedures developed specifically for the maintenance of fibroblast cells [23]. WI38 human fetal lung fibroblasts were obtained from Vincent J. Cristofalo and maintained in MEM as described above.

#### Chemicals

All chemicals were obtained from Sigma Aldrich (St. Louis, MO) unless otherwise noted.

#### DNA content analysis

Fibroblast cultures were seeded into 12-well tissue culture plates. The next day, colcemid (Invitrogen; Carlsbad, CA) was added to a final concentration of 70 ng/ml. After 48 or 72 hours, cells were trypsinized and washed in PBS, centrifuged at 500 × g for five minutes, and fixed in 70% ethanol overnight. DNA content was measured by staining with Guava Cell Cycle Reagent (Millipore; Billerica, MA) for 30 minutes and the percentage of polyploid (>4N DNA content) cells was measured as a fraction of the entire cycling population using the Guava EasyCyte Mini flow cytometer (Millipore).

## SUPPLEMENTAL FIGURES

Figure S1
