# Supplementary figures and images for: Relationship of spindle assembly checkpoint fidelity to species body mass, lifespan, and developmental rate
Source: Aging (Albany NY). 2011 Dec 26;3(12):1206–12. doi: 10.18632/aging.100416 (PMC3273901; doi:10.18632/aging.100416)

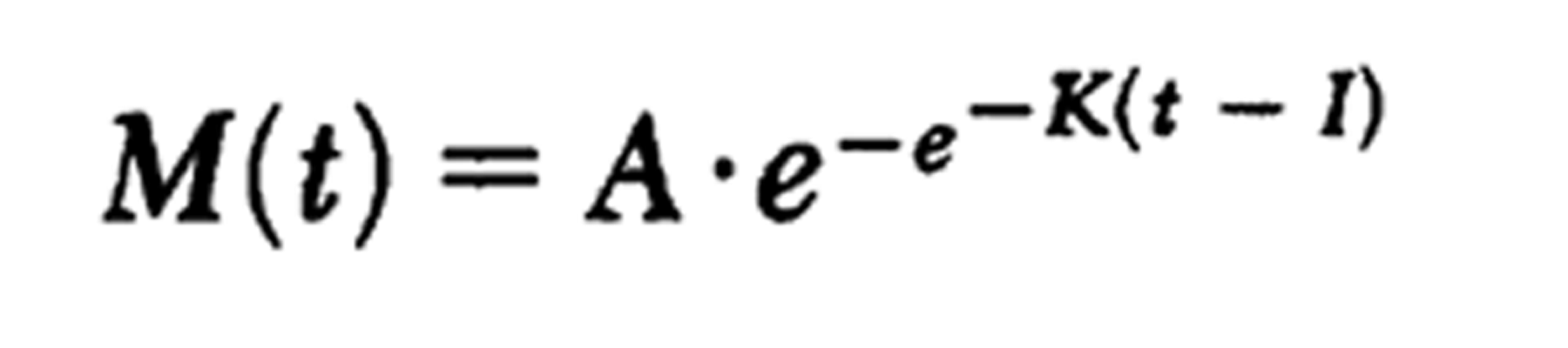

Supplement: Figure S1 [file aging-03-1206-s001.tif]
